# Benevolent Creativity Buffers Anxiety Aroused by Mortality Salience: Terror Management in COVID-19 Pandemic

**DOI:** 10.3389/fpsyg.2020.601027

**Published:** 2020-12-29

**Authors:** Yu-Xin Cui, Xiang Zhou, Chong Zu, Hong-Kun Zhai, Bo-Ren Bai, Yu-Mei Xu, Duo Li

**Affiliations:** ^1^Department of Social Psychology, Nankai University, Tianjin, China; ^2^Collaborative Innovation Center for China Economy, Tianjin, China

**Keywords:** mortality salience, benevolent creativity, malevolent creativity, anxiety, search for meaning, COVID-19

## Abstract

With the outbreak of the COVID-19 crisis, the public keeps getting epidemic-related information on the media. News reports on the increasing number of fatalities have exposed individuals to death, which causes negative emotional experiences such as tension, anxiety, and fear. This study aimed to investigate whether creativity could serve as an anxiety-buffer when mortality is salient. Based on previous findings, the present study utilized type of creative task and personal search for meaning as moderators. In Study 1, a 2 (mortality salience: absent, present) × 2 (type of creative task: benevolent, malevolent) between-subject design was utilized, and 168 subjects were randomly assigned to four experimental conditions. In Study 2, 221 subjects were recruited. The experimental procedure was similar to Study 1, except that the priming paradigm of mortality was changed and search for meaning was included as an additional moderating variable. State anxiety was measured as the dependent variable in both studies. Results of Study 1 showed that, while the benevolent creative task could buffer anxiety in the mortality salience condition, the malevolent creative task did not have the same effect. Furthermore, there was a significant interaction between mortality salience, type of creative task, and search for meaning in life on anxiety. In Study 2, the buffering function of benevolent creativity was more intense for participants with a higher level of search for meaning. Together, these findings reveal the influence of different types of creative tasks on individual anxiety levels under death priming conditions and the moderating effect of search for meaning in this relationship. Further, they suggest the need to focus on the role of creativity in terror management.

## Introduction

Since the end of 2019, the novel coronavirus epidemic has grown into a major global public health event. Owing to the declaration of the World Health Organization (WHO), the COVID-19 outbreak is characterized as a “pandemic” because it is increasingly spreading across the globe. This raging epidemic not only poses a great threat to public health and property but it also presents hidden risks to public mental health (Zhou, 2020). One of the major psychological threats associated with COVID-19 is terror and anxiety related to mortality (Courtney et al., 2020). From the perspective of the terror management theory, the present study aimed to investigate the role of creativity in buffering the anxiety caused by mortality salience, by distinguishing between benevolent and malevolent creativity. Furthermore, we explored whether individual differences in the level of search for meaning in life moderate the anxiety-buffering effect of creativity.

Like all animals, humans have a survival instinct; however, humans’ intellect makes them aware of their inevitable death. The conflict between this awareness and the motivation to survive has the potential to cause terrifying anxiety and a series of adverse effects (Greenberg et al., 2008; Juhl and Routledge, 2016). In everyday life, scenes, pictures, and words related to death (such as the death of a loved one, media coverage of death, or storylines involving death scenes on television) are likely to evoke deep fears of death. During the COVID-19 outbreak, individuals from all walks of life showed a passionate concern over this crisis. As the confirmed cases and deaths continued to increase, and the discussion about the epidemic became more widespread, the public was inevitably exposed to death-related information or situations. This, in turn, may activate their awareness of mortality and further evoke negative emotional outcomes such as anxiety and fear (Courtney et al., 2020). A survey of 515 Chinese residents showed that both anxiety and awareness of mortality were high during the COVID-19 epidemic (Fu, 2020). Song et al. (2020) analyzed data from 1,453 Chinese participants collected during the outbreak and found that death threats significantly increased individuals’ conformity consumption behavior. To manipulate perceived death threat, Ulqinaku et al. (2020) asked participants to recall the high morbidity and mortality of this novel coronavirus, and found that this perception influenced their unhealthy consumption behavior. Furthermore, mortality salience, and the emotional and behavioral responses it causes, have been examined extensively in other public emergencies such as Ebola (Arrowood et al., 2017), swine flu (Bélanger et al., 2013), food safety incidents (Li et al., 2015), and terrorist attacks (Das et al., 2009; Fischer-Pressler et al., 2019). Therefore, it is important to examine the effects of death threat during major public events such as the COVID-19 pandemic, and to identify ways to alleviate the anxiety caused by death awareness.

The terror management theory (Rosenblatt et al., 1989) was developed to explore how individuals regulate fear of death, and to explain the influence of death on emotion, cognition, and behavior. The theory suggests that, to escape the anxiety caused by mortality salience, individuals devise elaborate psychological defense mechanisms that help them obtain a sense of value and meaning, and a sense of symbolic immortality (Greenberg et al., 1994). Most studies have focused on two defenses: adherence to cultural worldview and self-esteem enhancement. On the one hand, reminders of mortality increase individuals’ need to espouse their cultural worldview. Previous research has found that awareness of death leads individuals to express more hostility toward those who violate their moral code and who criticize their culture (Greenberg et al., 1990; Florian and Mikulincer, 1997). On the other hand, mortality salience also promotes more self-esteem striving behaviors (Schmeichel et al., 2009) such as the tendency to estimate higher future financial success (Kasser and Sheldon, 2000) and holding fewer regrets about the past (Rudert et al., 2015). Some scholars also regard the search for a close relationship as a new defense mechanism, and they propose that investing in intimate relationships could buffer existential anxiety (Mikulincer et al., 2002). It’s worth mentioning that the above empirical findings are mainly based on the mortality salience hypothesis in terror management theory, which asserts that, if a psychological structure serves to buffer individuals from thoughts of their own death, priming individuals with death reminders would increase their reliance on that psychological structure (Florian et al., 2002). The buffering function of defense mechanisms on existential anxiety could also be examined from the perspective of the anxiety buffer hypothesis, which suggests that the activation of certain mechanisms in a mortality salience condition could satisfy the individual’s terror management need, and thus circumvent the impact of death threat. Studies have found that the prior threat or strengthening of worldview faith and self-esteem could moderate mortality salience effects (Schimel et al., 2007; Abeyta et al., 2014).

Creativity refers to the production of novel and practical ideas (Amabile, 1996). In most empirical research, creativity is viewed as an end goal and is studied as a dependent variable (Kaufman, 2018). However, with the expansion of the field of creativity research, scholars are increasingly concerned about the potential role of creativity in personal growth. For instance, researchers have found that creativity has a positive effect on coping with trauma (Forgeard, 2013; Forgeard et al., 2014) and on increasing sexual appeal (Lange and Euler, 2014). Previously, several studies have examined the role of creativity during mortality salience, but they reported conflicting findings. Arndt et al. (1999) conducted three sub-studies to understand the adverse psychological consequences of creative behavior when participants were reminded of death. Their results suggested that, following mortality salience priming, participants’ engagement in a creative task stimulated both guilt and an effort to reestablish social connections. They argued that creative action is always associated with breaking the routine, and therefore, it may be viewed as threatening to prevailing social norms, which do not conform to individuals’ pursuit after mortality salience. Therefore, completing a creative task might exacerbate mortality salience effects. Routledge (2005) also found that participating in creative tasks after mortality salience decreased individuals’ exploration tendency and they exhibited low openness to experience. In contrast, some studies suggest that creative behaviors could buffer the mortality salience effect. For instance, Routledge and Arndt (2009) found that participating in a creative task facilitated the exploration of cultural worldviews in response to thinking about death. Similarly, Xu et al. (2013) found that, following mortality salience manipulation, participants who wrote about past creative endeavors showed significantly lower death-thought accessibility than did those who wrote about past spending experiences. Based on previous research, it is evident that the relationship between creativity and the impact of death awareness may be influenced by a boundary condition. Therefore, the present study focused on the moderating function of creative behavior in the mortality salience effect by examining the role of benevolent and malevolent creativity separately.

Some studies have provided evidence for possible moderators in the role of creativity in terror management. For instance, Arndt et al. (2005) found that, following mortality salience, participants who engaged in a creative task experienced less guilt when they received feedback that emphasized their conformity as compared to when they received neutral feedback. Sligte et al. (2013) indicated that, when participants recognized legacy as the product of creativity, they showed more originality after being reminded of death as compared to when legacy was not involved. All these results suggest that when creativity is given extra meaning, or when the perception of creativity as being contrary to the mainstream social values is removed, it may serve as an anxiety-buffer during mortality salience.

Drawing upon previous research findings, the present study assumed that the valence of creativity may play an important role. Since the dark side of creativity was first outlined (McLaren, 1993), the topic of the valence of creativity has generally attracted public attention. In addition to being original and useful, creativity is also goal-oriented; hence, it may be directed toward either doing good or evil (Clark and James, 1999). Specifically, malevolent creativity refers to creativity generated for negative purposes, such as deliberate intent to harm others or damage others’ interests for self-benefit (Cropley et al., 2008), while benevolent creativity is associated with benevolent ends. According to the terror management theory, mortality salience increases the need for a meaningful self and for adherence to the cultural worldview, which could be achieved by participating in a benevolent rather than malevolent task. Especially in the Chinese cultural context, social connectedness is valued highly. Therefore, engaging in an interpersonal, friendly benevolent creative task could be viewed as a way to achieve meaning, thus buffering the anxiety aroused by death awareness. In contrast, malevolent creativity is generally regarded as an immoral behavior that is harmful to others. This could threaten inherent values and ethics, and could therefore increase anxiety in individuals experiencing mortality salience. Thus, we predicted that there would be a significant interaction between mortality salience and the type of creative task. Benevolent creativity, but not malevolent creativity, has an anxiety-buffering function in death-related conditions (Hypothesis 1).

Moreover, the mortality salience effect could also be moderated by personal search for meaning in life, which refers to the strength, intensity, and activity of individuals’ desire and efforts to establish and/or augment their understanding of the meaning, significance, and purpose of their life (Steger et al., 2008). Search for meaning is recognized as the main component of individual motivation, and it has been proven to have a moderating effect in the relationship between the presence of meaning and life satisfaction (Steger et al., 2011). Steger et al. (2011) further suggested that search for meaning behaves like a schema that could increase the salience of meaning-relevant information, and it provides original ways to understand individuals’ efforts to establish meaningful lives. If benevolent and malevolent creative tasks could be, respectively, regarded as a way to obtain or threaten meaning following mortality salience, their influence on anxiety may be moderated by search for meaning. Thus, we predicted that the impact of creative tasks on the mortality salience effect varies based on the level of search for meaning in life. The interaction between mortality salience and the type of creativity on anxiety is more intense in higher meaning-seeking individuals (Hypothesis 2).

## Study 1

In Study 1, we aimed to investigate whether different types of creativity could buffer the anxiety induced by exposure to news related to deaths during the COVID-19 epidemic.

### Methods

#### Participants

According to the calculations conducted using G^∗^Power 3.1 (Faul et al., 2007), a sample of at least 146 individuals is required to detect a moderate-size effect (*f* = 0.25) and power (1-β = 0.85). Considering the unstable factors in the experiments, we recruited 181 college students, of which 168 remained after deleting participants who did not read the materials as carefully as required (52 males and 116 females; ranging from undergraduates to doctoral candidates, *M*_age_ = 21.78 years, *SD*_age_ = 2.09 years).

#### Procedure and Materials

The experiment adopted a 2 (mortality salience: present, absent) × 2 (creative task: benevolent, malevolent) between-subjects design, and it was conducted online. Following a brief instruction about the experiment, participants were randomly assigned to two conditions for manipulating mortality salience (Das et al., 2009). In the mortality salience condition (henceforth referred to as “MS present condition”), participants read a news report about the death toll and subsequent outbreak trend of the COVID-19 epidemic. while in the MS absent condition, the news content was about the negative impact of COVID-19 on tourism. Both materials selected outbreak-related issues to better extract the impact of death awareness. Then, they were asked to write their feelings about this report and to answer a question related to the news to ensure that they had read it carefully.

Next, they completed a creative task (benevolent or malevolent), and evaluated the difficulty level and their level of goodwill or malice in this task using three 11-point Likert items (0 extremely low–10 extremely high). In the benevolent creative task, participants were told that the non-profit organization they administrated was experiencing a critical funding shortage, and they should come up with as many creative ideas for quickly raising money and helping the organization tide over this funding shortage. In the malevolent creative task, the organization was described as a profit-making company that was competing with one other company for acquiring a potential customer. As the customer had high moral requirements, if he knew that the other company’s president had once been an alcoholic, he would choose to partner with the participant’s company (see Appendix). Participants were asked to generate as many creative ways to subtly leak this information to the customer (Clark and James, 1999). Moreover, the creative task could also serve as a filler to lengthen the delay after the presentation of the news report, in order to better observe the distal effect of mortality salience. In the next step, participants completed the state anxiety scale (SAS) (Spielberg, 1983) as a measure of the dependent variable.

### Results and Discussion

The descriptive statistics of anxiety among four experimental groups are shown in Table 1. First, we conducted an independent sample *t*-test to check for the manipulation effectiveness of benevolent and malevolent creative tasks. Analyses showed that participants in the benevolent creativity group perceived more kindness (*M* = 8.70, *SD* = 1.80) than did those in the malevolent group (*M* = 5.02, *SD* = 2.33), *t*(166) = 11.435, *p* < 0.001, cohen’s *d* = 1.765. Meanwhile, participants in the benevolent creativity group showed significant lower perception of malice (*M* = 2.54, *SD* = 1.55) as compared to those in the malevolent group (*M* = 7.15, *SD* = 2.05), *t*(166) = −16.477, *p* < 0.001, cohen’s *d* = −2.543. On applying the Bonferroni correction, the cutoff for both significance levels were still less than 0.001. The results indicated that the manipulation of the type of creative task was effective. For the manipulation check pertaining to mortality salience, we conducted a word frequency analysis of participants’ thoughts on the news through ROST 6.0, and counted 20 most frequently occurring words (Klackl and Jonas, 2019). Participants in the MS present group use more words related to death (e.g., life, virus, death, fatality rate, infection), while those in the MS absent group regularly used death-unrelated words (e.g., tourism, economy, industry). This suggests that the manipulation of mortality salience was successful, and that the news in the MS present condition provoked more thoughts about death. There was no significant difference in anxiety scores between male and female participants, *t*(166) = −0.683, *p* = 0.496, cohen’s *d* = −0.114, thus, gender was not included in the follow-up analysis.

**TABLE 1:** Descriptive statistics of anxiety scores under different experimental conditions.

	MS-absent	MS-present
**Benevolent creative task**		
*M*	41.70	36.90
*SD*	9.34	11.38
*n*	43	41
**Malevolent creative task**		
*M*	39.24	44.88
*SD*	9.71	10.98
*n*	42	42

**FIGURE 1:**
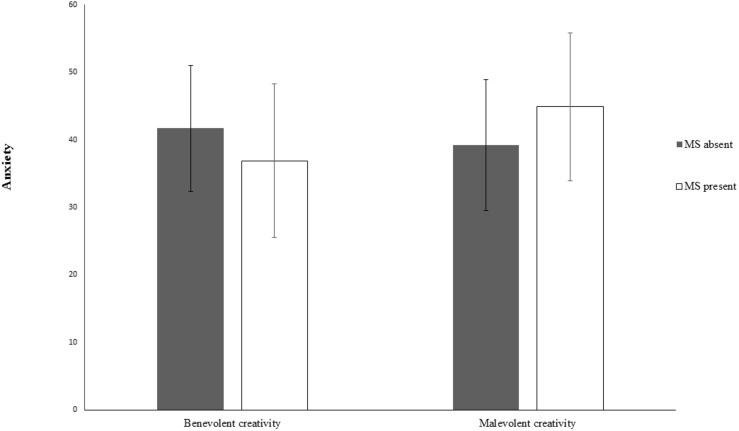
Interaction between mortality salience and type of creativity (Study 1).

Then, a two-way analysis of variance (ANOVA) was conducted on the anxiety level of participants in different manipulation conditions, with perceived difficulty of the creative task as a covariate. There was no significant main effect of mortality salience, *F*(1,163) = 0.137, *p* = 0.712, or the type of creative task, *F*(1, 163) = 2.551, *p* = 0.112. As predicted, the interaction between the two independent variables was significant, *F*(1,163) = 10.621, *p* = 0.001, η_p_^2^ = 0.061, 1-β = 0.900. The simple effect analysis revealed that, in the benevolent condition, the MS present group reported less anxiety than did those in the control group, *F*(1,163) = 4.167, *p* = 0.043, η_p_^2^ = 0.025. In the malevolent condition, the MS present group reported more anxiety than did those in the control group, *F*(1,163) = 6.588, *p* = 0.011, η_p_^2^ = 0.039 (see Figure 1).

Thus, it appears that after the activation of mortality salience by reading specific news reports, creativity could serve as an anxiety-buffer when it was endowed with positive intentions. However, creative tasks that aimed to gain an unfair advantage or damage others did not serve such a function. To some degree, this result is in accordance with a previous finding that amplified concerns about mortality inhibited self-directed creativity but promoted community-directed creativity (Routledge et al., 2008).

## Study 2

Study 1 verified that reading the news report about the death toll of COVID-19 could activate mortality salience in participants, and that the valence of creativity interacts with mortality salience. However, irrelevant variables in news materials were not controlled strictly in this experiment. To further clarify the role of creativity in the mortality salience effect, Study 2 conducted a typical manipulation of mortality salience and introduced search for meaning as an additional moderating variable.

### Method

#### Participants

From previous research, the effect size of three-way interaction related to mortality salience is 0.04 (Perach and Wisman, 2016). According to the calculations conducted using G^∗^Power 3.1, a sample of at least 199 is required (*f*^2^ = 0.04, 1-β = 0.8). A total of 234 college students were recruited online. For testing whether the participants carefully response to the items, we adopted a trap question (e.g., please choose “absolutely not” for this item). After excluding participants who did not pass the trap question, the final sample size for this study was 221 (73 males, 148 females; ranging from undergraduates to doctoral candidates, *M*_age_ = 22.72 years, *SD*_age_ = 3.94 years). Each participant was randomly assigned to one of four conditions and was paid at the end of the experiment.

#### Procedure and Materials

This online study applied the same 2 (mortality salience: present, absent) × 2 (creative task: benevolent, malevolent) design and experimental procedure as in Study 1, but there were two changes. First, we turned to a typical paradigm for mortality salience priming (Rosenblatt et al., 1989). The MS present group required participants to respond to the following two open-ended questions: “Please briefly describe the emotions that the thought of your own death arouses in you” and “Jot down, as specifically as you can, what you think will happen to you physically as you die and once you are physically dead.” Participants in MS absent group were asked, “Please briefly describe the emotions that the thought of your own dental pain arouses in you” and “Jot down, as specifically as you can, what you think will happen to you physically when you are experiencing dental pain.”

Second, we introduced search for meaning in life as a moderating variable, and measured it using the Chinese version of the Meaning in Life Questionnaire-Search (MLQ-S) (Steger et al., 2006; Gan and Liu, 2010), which consists of four items (Cronbach’s α = 0.872 in the present study). The scale includes items such as “I’m looking for something that makes my life feel meaningful.” Each question is rated on a seven-point Likert scale ranging from absolutely untrue to absolutely true.

### Results and Discussion

As in study 1, the word frequency analysis was conducted for the manipulation check of mortality salience. Participants in the MS present group write more words related to death (e.g., body, death, stiff, soul), while those in the MS absent group used more dental pain-related words (e.g., toothache, teeth, pain, dentist). The results suggested that the manipulation of mortality salience was successful. Moreover, there was no significant difference in anxiety (*t*(219) = −0.773, *p* = 0.440, cohen’s *d* = −0.111)and search for meaning in life (*t*(219) = −0.467, *p* = 0.641, cohen’s *d* = −0.067) between male and female participants, thus, gender was not included in follow-up studies.

We coded mortality salience (absent = 0, present = 1) and creative tasks (benevolent = 0, malevolent = 1). To analyze the effect of mortality salience, valence of creativity, and search for meaning in life on anxiety, we conducted a hierarchical linear regression. In the first step, difficulty (centered) was entered as a control variable; in the second step, mortality salience (dummy coded), type of creative task (dummy coded), and search for meaning in life (centered) were entered the regression. In the third step, we added the two-way interaction, and in the final step, the three-way interaction was included in the regression. As shown in Table 2, in the second step, the main effect of mortality salience (β = 0.014, *t* = 0.229, *p* > 0.05) and search for meaning in life (β = −0.064, *t* = −1.039, *p* > 0.05) were not significant, the main effect of valence of creativity was significant (β = 0.192, *t* = 3.091, *p* = 0.002). In the third step, no two-way interaction was significant. In the fourth step, as predicted, the inclusion of the three-way interaction increased the power of the model in explaining the variance in anxiety (Δ*R*^2^ = 0.025, *p* = 0.009), and there was a significant interaction between mortality salience, creative task, and search for meaning in life (β = 0.384, *t* = 2.630, *p* = 0.009).

**TABLE 2:** Results of the hierarchical regression (Study 2).

Predictors	Step 1		Step 2		Step 3		Step 4	
	β	*SE*	β	*SE*	β	*SE*	β	*SE*
Constant		0.71		1.21		1.40		1.38
Difficulty	0.41***	0.34	0.37***	0.34	0.38***	0.35	0.36***	0.34
MS (present = 1)			0.01	1.41	−0.02	1.97	−0.01	1.94
CT (malevolent = 1)			0.19**	1.43	0.16	2.06	0.14	2.03
SML			−0.06	0.18	0.02	0.32	0.20	0.37
MS × CT					0.06	2.83	0.06	2.79
MS × SML					−0.07	0.36	−0.34*	0.54
CT × SML					−0.04	0.36	−0.30*	0.52
MS × CT × SML							0.38**	0.72
*R*^2^	0.165		0.207		0.211		0.236	
Δ*R*^2^	0.165		0.042		0.004		0.025	
Δ*F*	43.302		3.772*		0.384		6.918**	

MS, mortality salience; CT, creative task; SML, search for meaning in life. *p < 0.05, **p < 0.01; **p < 0.001.

**FIGURE 2:**
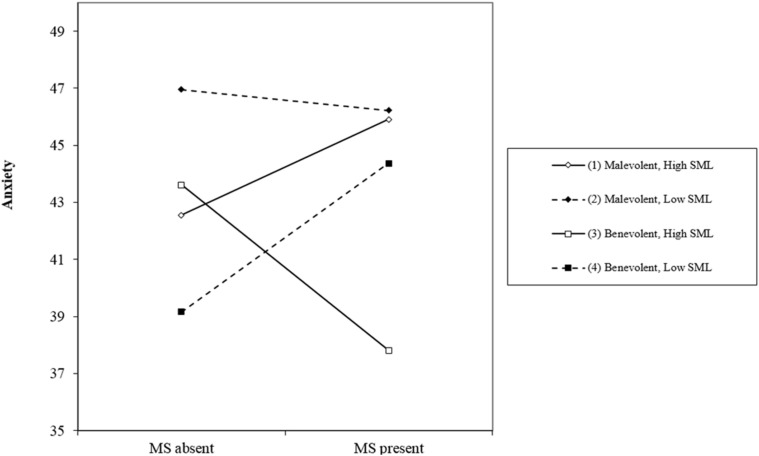
Three-way interaction between mortality salience (MS), creative task and search for meaning (SML) on anxiety, after controlling for difficulty (Study 2).

To further examine the three-way interaction, we applied the SPSS PROCESS macro on Model 3 (three-way interaction) (Hayes, 2013). Figure 2 illustrates the interaction between the three variables on controlling for difficulty. The MS × CT interaction was significant only in participants with a higher level of search for meaning in life (+*SD*), *B* = 9.128, *t* = 2.287, *p* = 0.023, 95% confidence interval (CI) [1.258, 16.997], and for these participants, completing a benevolent creative task could significantly buffer the anxiety experienced in the MS present condition, *B* = −5.639, *t* = −1.998, *p* = 0.047, 95%CI [−11.202, −0.076]. For the participants with lower search for meaning (−*SD*), the interaction between mortality salience and creative task no longer existed, *B* = −5.924, *t* = −1.482, *p* > 0.05, 95%CI [−13.802, 1.953].

These results supported Hypothesis 2 and provided additional evidence for the existential buffering function of benevolent creativity and for the role of search for meaning as a boundary condition. The anxiety-buffering effect of benevolent creativity was not observed in all participants. Only in those with a higher level of search for meaning in life, completing a benevolent creative task circumvented the anxiety aroused by mortality salience. Further, completing a malevolent creative task intensified the mortality salience effect. Hofer (2013) also found a similar result, that for participants with high search for meaning in life, mortality salience increased appreciation of a meaningful film in comparison to the control group.

## General Discussion

The present research incorporated the current context of COVID-19 and examined the role of creativity in the mortality salience effect within the framework of the terror management theory. Overall, the results of two studies support the hypothesis that the moderating role of creativity in the mortality salience effect depends on its valence and on the level of personal search for meaning. The first study applied COVID-19-related news reports to manipulate mortality salience to simulate real-life exposure to information about death. The results showed that benevolent creativity could buffer the anxiety aroused by reading a death-related news report, while malevolent creativity could not. In Study 2, we replaced the death awareness priming paradigm with classic priming, which was more commonly utilized, to focus more precisely on the mortality salience effect. Additionally, we included search for meaning in life as a moderator to explore its interaction with mortality salience and valence of creative tasks on state anxiety. Results revealed that, only among participants with high levels of search for meaning, the benevolent creative task was associated with reduced anxiety in the MS present condition as compared to the control condition.

Our findings are partially consistent with the assumption of the terror management theory, and the views of Arndt et al. (2005) and Sligte et al. (2013), that creativity emphasizes self-pursuit and breaking the routine, which is not in accordance with the need for the maintenance of social bonds and faith in an inherent cultural worldview following mortality salience. However, when this rebellious view of creativity is removed (e.g., the goal of creative behavior is in line with mainstream values or is beneficial for maintaining social ties) or creativity is assigned extra meaning (e.g., a legacy), engaging in creative tasks no longer threatens these needs. Instead, it serves as a means of obtaining meaning to buffer the anxiety-arousing effects of mortality salience. This might explain why benevolent creativity has an anxiety-buffer function following mortality salience and malevolent creativity does not.

Furthermore, the present findings on the effect of searching for meaning are in line with the moderating role reported by Hofer (2013). It is plausible that individuals who have an intense tendency to search for meaning in life may perceive the meaning in creative activities more accurately, and thus, they would utilize it to protect themselves from the anxiety rooted in the contemplations of mortality. On the other hand, individuals with low search for meaning in life would be more insensitive when facing the mortality reminder or the creative task, which leads to a non-significant interaction between mortality salience and type of creative task on anxiety.

### Theoretical and Practical Implications

In general, the findings of this study add to the existing literature on the terror management theory and creativity in several ways. First, creativity is often regarded as an outcome variable, and previous research has mostly focused on the influence of environmental or personal factors on creative performance. As Kaufman (2018) proposed, besides understanding how individuals can become creative, future empirical studies also need to examine how creativity can help them achieve positive outcomes. The present study explored the role of creativity in buffering the anxiety caused by mortality salience, and provided empirical evidence for understanding the positive role of creativity in personal growth, and in maintaining physical and mental health. Furthermore, considering previous controversial findings about the role of creativity in mortality salience, this study introduced the valence of creativity and search for meaning in life as moderator variables to clarify the boundary conditions involved in this relationship. The results provide a new perspective that explains the inconsistent findings obtained by previous studies, and further contribute to research on terror management.

The present study also has several practical implications. The spread of COVID-19 may activate individuals’ awareness of death and further trigger a series of emotional, cognitive, and behavioral responses. Based on this reality, the present study aimed to explore whether creative behavior could serve an anxiety-buffering function in the context of mortality salience by distinguishing creative tasks as benevolent and malevolent. Our results not only provide important implications for developing personal outbreak responses and collective psychological interventions during the COVID-19 epidemic, but also provide a theoretical basis for future responses to major public emergencies. The findings suggest that, when individuals experience a death threat, participation in benevolent creative activities may provide meaning and value, which may in turn reduce the anxiety evoked by mortality salience. Moreover, personal traits should also be considered when discussing the role of creativity in the mortality salience effect. In the present study, after initiating death awareness, benevolent creativity showed a stronger anxiety buffer effect for participants with a high level of search for meaning in life. These results indicate that individual characteristics should be considered carefully while developing psychological services, which may improve their effectiveness.

### Limitations and Prospects

Although this study explored the role of mortality salience, valence of creative tasks, and search for meaning in life on anxiety, it had several limitations. First, all participants in the study were college students; further studies could explore the role of creativity in the mortality salience effect among a broader group of participants, such as the elderly or children, to increase the generalizability of the present results. Second, the main effect of death awareness on anxiety was not significant in both studies. This finding may have been influenced by the countervailing effect of benevolent and malevolent creativity. In future studies, a control group without a creative task could be added to derive a better explanation for this result. Third, in the present study, the role of creative process, rather than creative outcome in terror management was concerned. Another direction for future study is to consider the mortality salience’s effect on the performance of benevolent and malevolent creativity, to further discuss how the valence of creativity matters in terror management. As for the content of the creative task, it’s also necessary to develop more real-world problem-solving tasks to cover a variety of benevolent and malevolent situations in life. Last but not the least, the present study chose anxiety as the dependent variable, but the outcome variables of mortality salience are by no means limited to anxiety. Future studies could investigate the impact of creativity on the mortality salience effect from a variety of research perspectives, such as customer behavior, health-related behavior, and decision-making. Future research could also examine the role of collaborative creative activities.

## Data Availability Statement

The datasets generated for this study are available on request to the corresponding author.

## Ethics Statement

The studies involving human participants were reviewed and approved by Institutional Review Board of Psychology of Nankai University. The patients/participants provided their written informed consent to participate in this study.

## Author Contributions

Y-XC, CZ, XZ, and H-KZ developed the research idea together. Y-XC collected and analyzed the data with the assistance of B-RB and XZ, and drafted the manuscript. Y-MX, DL, and B-RB provided critical revisions. All authors contributed to the article and approved the submitted version.

## Conflict of Interest

The authors declare that the research was conducted in the absence of any commercial or financial relationships that could be construed as a potential conflict of interest.

## Appendix

### Instructions for the Creative tasks

Benevolent creativity: Please imagine that you are in position of authority in a non-profit organization. Recently, the organization is experiencing a serious shortage of funds and can hardly afford activities. Only when sufficient funds are raised as soon as possible, can the organization meet its obligations and complete ongoing projects, so as to better serve the public interest. How to quickly raise funds for the organization in a short time? Please generate as many creative ways as you can (the plans don’t need to be written in detail, just express them clearly).

Malevolent creativity: Please imagine that you are in position of authority in a profit-making organization. The organization is now competing fiercely with one other company for a potential customer. The customer is a morally focused person, and if he knows the other company’s president had once been an alcoholic, he would award the contract to your organization. However, the point is not to let the customer know that you leaked this information. How to tell the customer about your competitor’s drinking problem without exposing yourself? Please generate as many creative ways as you can (the plans don’t need to be written in detail, just express them clearly).

## References

[B1] Abeyta A. A. Juhl J. Routledge C. (2014). Exploring the effects of self-esteem and mortality salience on proximal and distally measured death anxiety: a further test of the dual process model of terror management. *Motiv. Emot.* 38 523–528. 10.1007/s11031-014-9400-y

[B2] Amabile T. M. (1996). *Creativity in Context: Update to the Social Psychology of Creativity.* Boulder, CO: Westview press.

[B3] Arndt J. Greenberg J. Solomon S. Pyszczynski T. Schimel J. (1999). Creativity and terror management: evidence that creative activity increases guilt and social projection following mortality salience. *J. Pers. Soc. Psychol.* 77 19–32. 10.1037/0022-3514.77.1.19 10573872

[B4] Arndt J. Routledge C. Greenberg J. Sheldon K. M. (2005). Illuminating the dark side of creative expression: assimilation needs and the consequences of creative action following mortality salience. *Pers. Soc. Psychol. Bull.* 31 1327–1339. 10.1177/0146167205274690 16143665

[B5] Arrowood R. B. Cox C. R. Kersten M. Routledge C. Shelton J. T. Hood R. W. (2017). Ebola salience, death-thought accessibility, and worldview defense: a terror management theory perspective. *Death Stud.* 41 585–591. 10.1080/07481187.2017.1322644 28436743

[B6] Bélanger J. J. Faber T. Gelfand M. J. (2013). Supersize my identity: when thoughts of contracting swine flu boost one’s patriotic identity. *J. Appl. Soc. Psychol.* 43 E153–E155. 10.1111/jasp.12032

[B7] Clark K. James K. (1999). Justice and positive and negative creativity. *Creat. Res. J.* 12 311–320. 10.1207/s15326934crj1204_9

[B8] Courtney E. P. Goldenberg J. L. Boyd P. (2020). The contagion of mortality: a terror management health model for pandemics. *Br. J. Soc. Psychol.* 59 607–617. 10.1111/bjso.12392 32557684 PMC7323320

[B9] Cropley D. H. Kaufman J. C. Cropley A. J. (2008). Malevolent creativity: a functional model of creativity in terrorism and crime. *Creat. Res. J.* 20 105–115. 10.1080/10400410802059424

[B10] Das E. Bushman B. J. Bezemer M. D. Kerkhof P. Vermeulen I. E. (2009). How terrorism news reports increase prejudice against outgroups: a terror management account. *J. Exp. Soc. Psychol.* 45 453–459. 10.1016/j.jesp.2008.12.001

[B11] Faul F. Erdfelder E. Lang A. G. Buchner A. (2007). G^∗^power 3: a flexible statistical power analysis program for the social, behavioral, and biomedical sciences. *Behav. Res. Methods* 39 175–191. 10.3758/BF03193146 17695343

[B12] Fischer-Pressler D. Schwemmer C. Fischbach K. (2019). Collective sense-making in times of crisis: connecting terror management theory with twitter user reactions to the berlin terrorist attack. *Comput. Human Behav.* 100 138–151. 10.1016/j.chb.2019.05.012

[B13] Florian V. Mikulincer M. (1997). Fear of death and the judgment of social transgressions: a multidimensional test of terror management theory. *J. Pers. Soc. Psychol.* 73 369–380. 10.1037//0022-3514.73.2.369 9248054

[B14] Florian V. Mikulincer M. Hirschberger G. (2002). The anxiety-buffering function of close relationships: evidence that relationship commitment acts as a terror management mechanism. *J. Pers. Soc. Psychol.* 82 527–542. 10.1037/0022-3514.82.4.527 11999922

[B15] Forgeard M. J. C. (2013). Perceiving benefits after adversity: the relationship between self-reported posttraumatic growth and creativity. *Psychol. Aesthet. Creat. Arts* 7 245–264. 10.1037/a0031223

[B16] Forgeard M. J. C. Mecklenburg A. C. Lacasse J. J. Jayawickreme E. (2014). “Bringing the whole universe to order: creativity, healing, and posttraumatic growth,” in *Creativity and Mental Illness*, ed. Kaufman J. C. (New York, NY: Cambridge University Press), 321–342. 10.1017/cbo9781139128902.021

[B17] Fu L. (2020). Public psychological characteristics and intervention strategies in emergencies: a survey during the COVID-19 epidemic. *Stud. Ideol. Educ.* 27, 60–65.

[B18] Gan Y. Liu S. (2010). Reliability and validity of the Chinese version of the meaning in life questionnaire. *Chin. J. Clin. Psychol.* 24 478–482. 10.3969/j.issn.1000-6729.2010.06.021

[B19] Greenberg J. Pyszczynski T. Solomon S. Rosenblatt A. Veeder M. Kirkland S. (1990). Evidence for terror management theory II: the effects of mortality salience on reactions to those who threaten or bolster the cultural worldview. *J. Pers. Soc. Psychol.* 58 308–318. 10.1037/0022-3514.58.2.308

[B20] Greenberg J. Pyszczynski T. Solomon S. Simon L. Breus M. (1994). Role of consciousness and accessibility of death-related thoughts in mortality salience effects. *J. Pers. Soc. Psychol.* 67 627–637. 10.1037/0022-3514.67.4.627 7965609

[B21] Greenberg J. Solomon S. Arndt J. (2008). “A basic but uniquely human motivation: terror management,” in *Handbook of Motivation Science*, eds Shah J. Y. Gardner W. L. (New York, NY: The Guilford Press), 114–134.

[B22] Hayes A. F. (2013). *Introduction to Mediation, Moderation, and Conditional Process Analysis: A Regression-Based Approach.* New York, NY: Guilford Press.

[B23] Hofer M. (2013). Appreciation and enjoyment of meaningful entertainment: the role of mortality salience and search for meaning in life. *J. Media Psychol. Theor. Methods Appl.* 25 109–117. 10.1027/1864-1105/a000089

[B24] Juhl J. Routledge C. (2016). Putting the terror in terror management theory: evidence that the awareness of death does cause anxiety and undermine psychological well-being. *Curr. Dir. Psychol. Sci.* 25 99–103. 10.1177/0963721415625218

[B25] Kasser T. Sheldon K. M. (2000). Of wealth and death: materialism, mortality salience, and consumption behavior. *Psychol. Sci.* 11 348–351. 10.1111/1467-9280.00269 11273398

[B26] Kaufman J. C. (2018). Finding meaning with creativity in the past, present, and future. *Perspect. Psychol. Sci.* 13 734–749. 10.1177/1745691618771981 30227083

[B27] Klackl J. Jonas E. (2019). Effects of mortality salience on physiological arousal. *Front. Psychol.* 10:1893. 10.3389/fpsyg.2019.01893 31481914 PMC6710453

[B28] Lange B. P. Euler H. A. (2014). Writers have groupies, too: high quality literature production and mating success. *Evol. Behav. Sci.* 8 20–30. 10.1037/h0097246

[B29] Li Y. Liu M. Ping Y. (2015). Research on consumers’ purchase intention fluctuation after food safety incident based on terror management dual-process perspective. *Manag. Rev.* 27 186–196.

[B30] McLaren R. B. (1993). The dark side of creativity. *Creat. Res. J.* 6 137–144. 10.1080/10400419309534472

[B31] Mikulincer M. Florian V. Birnbaum G. Malishkevich S. (2002). The death-anxiety buffering function of close relationships: exploring the effects of separation reminders on death-thought accessibility. *Pers. Soc. Psychol. Bull.* 28 287–299. 10.1177/0146167202286001

[B32] Perach R. Wisman A. (2016). Can creativity beat death? A review and evidence on the existential anxiety buffering functions of creative achievement. *J. Creat. Behav.* 53 193–210. 10.1002/jocb.171

[B33] Rosenblatt A. Greenberg J. Solomon S. Pyszczynski T. Lyon D. (1989). Evidence for terror management theory I: the effects of mortality salience on reactions to those who violate or uphold cultural values. *J. Pers. Soc. Psychol.* 57 681–690. 10.1037/0022-3514.57.4.681 2795438

[B34] Routledge C. (2005). *To Boldly Go Where No One has Gone Before: The Impact of Creativity and Openness to Experience on Exploration After Mortality Salience.* Unpublished Doctorial dissertation, University of Missouri-Columbia, Columbia, MO.

[B35] Routledge C. Arndt J. Vess M. Sheldon K. M. (2008). The life and death of creativity: the effects of mortality salience on self versus social-directed creative expression. *Motiv. Emot.* 32 331–338. 10.1007/s11031-008-9108-y

[B36] Routledge C. D. Arndt J. (2009). Creative terror management: creativity as a facilitator of cultural exploration after mortality salience. *Pers. Soc. Psychol. Bull.* 35 493–505. 10.1177/0146167208329629 19139160

[B37] Rudert S. C. Reutner L. Walker M. Greifeneder R. (2015). An unscathed past in the face of death: mortality salience reduces individuals’ regrets. *J. Exp. Soc. Psychol.* 58 34–41. 10.1016/j.jesp.2014.12.006

[B38] Schimel J. Hayes J. Williams T. Jahrig J. (2007). Is death really the worm at the core? Converging evidence that worldview threat increases death-thought accessibility. *J. Pers. Soc. Psychol.* 92 789–803. 10.1037/0022-3514.92.5.789 17484605

[B39] Schmeichel B. J. Gailliot M. T. Filardo E. A. McGregor I. Gitter S. Baumeister R. F. (2009). Terror management theory and self-esteem revisited: the roles of implicit and explicit self-esteem in mortality salience effects. *J. Pers. Soc. Psychol.* 96 1077–1087. 10.1037/a0015091 19379037

[B40] Sligte D. J. Nijstad B. A. De Dreu C. K. W. (2013). Leaving a legacy neutralizes negative effects of death anxiety on creativity. *Pers. Soc. Psychol. Bull.* 39 1152–1163. 10.1177/0146167213490804 23861202

[B41] Song W. Jin X. Gao J. Zhao T. (2020). Will buying follow others ease their threat of death? An analysis of consumer data during the period of COVID-19 in China. *Int. J. Environ. Res. Public Health* 17:3215. 10.3390/ijerph17093215 32384598 PMC7246543

[B42] Spielberg C. D. (1983). *Manual for the State-Trait Anxiety Inventory.* Palo Alto, CA: Consulting Psychologists Press.

[B43] Steger M. F. Frazier P. Oishi S. Kaler M. (2006). The meaning in life questionnaire: assessing the presence of and search for meaning in life. *J. Couns. Psychol.* 53 80–93. 10.1037/0022-0167.53.1.80

[B44] Steger M. F. Kashdan T. B. Sullivan B. A. Lorentz D. (2008). Understanding the search for meaning in life: personality, cognitive style, and the dynamic between seeking and experiencing meaning. *J. Pers.* 76 199–228. 10.1111/j.1467-6494.2007.00484.x 18331281

[B45] Steger M. F. Oishi S. Kesebir S. (2011). Is a life without meaning satisfying? The moderating role of the search for meaning in satisfaction with life judgments. *J. Posit. Psychol.* 6 173–180. 10.1080/17439760.2011.569171

[B46] Ulqinaku A. Sarial−Abi G. Kinsella E. L. (2020). Benefits of heroes to coping with mortality threats by providing perceptions of personal power and reducing unhealthy compensatory consumption. *Psychol. Mark.* 37 1433–1445. 10.1002/mar.21391 32836727 PMC7405095

[B47] Xu H. Brucks M. L. Guo L. (2013). Creative consumption after mortality salience: compared to what, for whom, what tasks? And a time horizon issue. *J. Res. Consum.* 24 1–5.

[B48] Zhou X. (2020). Psychological guidance in dynamically coping with COVID-19. *Peoples Trib.* 29 32–35.

